# Seed Transcriptomics Analysis in *Camellia oleifera* Uncovers Genes Associated with Oil Content and Fatty Acid Composition

**DOI:** 10.3390/ijms19010118

**Published:** 2018-01-02

**Authors:** Ping Lin, Kailiang Wang, Changfu Zhou, Yunhai Xie, Xiaohua Yao, Hengfu Yin

**Affiliations:** 1 Research Institute of Subtropical Forestry, Chinese Academy of Forestry, Hangzhou 311400, China; linping80@126.com (P.L.); wangkl163@163.com (K.W.); 2 Research Institute of Horticulture, Hunan Academy of Agricultural Sciences, Changsha 410125, China; zhouchangfu555@126.com; 3 Forestry Seedling Management Station of Zhejiang Province, Hangzhou 310020, China; yunhaix@126.com

**Keywords:** *Camellia oleifera*, transcriptomics, lipid biosynthesis, fatty acid, seed oil

## Abstract

*Camellia oleifera* is a major tree species for producing edible oil. Its seed oil is well known for the high level of oleic acids; however, little is known regarding the molecular mechanism of lipid biosynthesis in *C. oleifera*. Here, we measured the oil contents and fatty acid (FA) compositions at four developmental stages and investigated the global gene expression profiles through transcriptomics sequencing. We identified differentially-expressed genes (DEGs) among the developmental stages and found that the distribution of numbers of DEGs was associated with the accumulation pattern of seed oil. Gene Ontology (GO) enrichment analysis revealed some critical biological processes related to oil accumulation, including lipid metabolism and phosphatidylcholine metabolism. Furthermore, we investigated the expression patterns of lipid biosynthesis genes. We showed that most of the genes were identified with single or multiple copies, and some had correlated profiles along oil accumulation. We proposed that the higher levels of *stearoyl-ACP desaturases* (*SADs*) coupled with lower activities of *fatty acid desaturase 2* (*FAD2*) might be responsive to the boost of oleic acid at the late stage of *C. oleifera* seeds’ development. This work presents a comprehensive transcriptomics study of *C. oleifera* seeds and uncovers valuable DEGs that are associated with the seed oil accumulation.

## 1. Introduction

*Camellia oleifera* originates from China and is planted widely in south China with a long cultivation history of over 2000 years. As one of the four major oil trees, together with oil palm, olive and coconut, it is known as an important edible oil-bearing tree species in China. It was reported in 2015 that the dry seed production of oil was around ~2.2 million tons over a cultivated area of 4 million ha. *Camellia* oil, the major product of *C. oleifera* seeds (nearly 50% of dry kernel weight), is considered to be among the highest quality oils [1,2]. The unsaturated FAs account for approximately 90% of all FAs, including monounsaturated fatty acid (MUFA), oleic acid (18:1), and polyunsaturated fatty acid (PUFA), linoleic acid (18:2) mainly [3]. *C. oleifera* has received much more attention owing to its high unsaturated FA contents, which are good for human health. It is also widely used in cosmetics, ink, lubricants, etc. [4,5].

The biochemistry of lipid biosynthesis has been extensively studied in many plants [6,7]. It normally includes three parts in plant cells: de novo FA synthesis in plastids, FA modification, acyl editing and triacylglycerol (TAG) assembly in endoplasmic reticulum [6,8]. The de novo synthesis of FA is initiated in the plastid by acetyl-CoA carboxylase (ACCase) converting acetyl-CoA to malonyl-CoA; diverse FA, including PUFA and part of the long chain FA (carbon chain length over 18), are produced by FA modification and acyl editing in endoplasmic reticulum, and oil body is formed and accumulated at the TAG assembly ending stage [8]. Some studies indicate that the TAG accumulation is limited by FA production in plastid [9,10]. The plastid ACCase is the key rate-determining enzyme to control FA synthesis and is regulated by transcription factors [11,12,13]. Stearoyl-ACP desaturases (SADs) in plastid are considered as key enzymes for FA desaturation, which introduce the first double bound between carbon 9 and 10 [14,15], and the Δ^12^(ω^6^)-desaturase is one of the key rate-determining enzymes for the PUFA synthesis in some oil crops [16,17]. Another study’s results show that the TAG assembly is also important to control the lipid accumulation in developing oilseed, such as that in oilseed rape [18], soybean [19], tung tree [20], etc. The last step of TAG assembly is catalyzed by acyl-CoA: diacylglycerol (DAG) acyltransferase (DGAT), and ectopic expression of *DGATs* in *Glycine max* and *Brassica napus* significantly increased the seed oil contents [19,21].

In many oil crops, the high oil production is synthesized in seed kernels, such as soybean [22], hickory [23] and rapeseed [24]; while in oil palm, the oil production was accumulated in fruit tissue mesocarp [25]. The seed oil of *C. oleifera* was dominant with 18:1, which was similar to hickory and olive [23,26]. Huang and coauthors showed that the expression of a high level of *SAD* with a low level of *FAD2* was associated with the 18:1 synthesis in hickory [23]. In olive, the genomic characterization revealed that a newly-evolved small RNA, which repressed the expression of *FAD2*, contributed to high levels of 18:1 content [26]. In recent years, multiple *Camellia* sp. transcriptomes were characterized separately to identify major biology processes and genes involved in tea-specific compounds synthesis [27], cold acclimation [28], response to natural drying of seed [29], drought stress [30], etc. Some genes involved in oil accumulation and FA composition during the *C. oleifera* seeds’ development have been studied including *fructose-1,6-bisphosphate aldolase* genes [31], *SAD* [32] and *Δ-12 desaturase* [33]. However, the understanding of the molecular regulation of oil accumulation and FA composition in *C. oleifera* developing seeds remains limited.

With the increasing demands for healthy edible oil, the production of *Camellia* oil cannot meet the requirements of the market. Increasing the yield and improving the quality are the major challenges for the *Camellia* oil industry. To identify genes related to lipid biosynthesis in *C. oleifera* seeds, we generated a comprehensive transcriptome of kernel at four developmental stages displaying substantial changes of oil contents and compositions. We identified six sets of differentially-expressed genes (DEGs) during the oil accumulation in kernel and found that the distribution of the numbers of DEGs was associated with the oil accumulation pattern. Furthermore, expression analysis of key enzymes of lipid biosynthesis revealed that the regulation of *SADs* and fatty acid desaturase 2 (*FAD2*) levels at the late stages of seed development might be a critical transition for 18:1 accumulation. This work provides insights into the regulation of seed oil biosynthesis in *C. oleifera* and is valuable to the genetic breeding program towards the production of high quality and yield of *Camellia* oil.

## 2. Results

### 2.1. Characterizations of Oil Content and Fatty Acid (FA) Composition during Seed Development of C. oleifera

**Figure 1:**
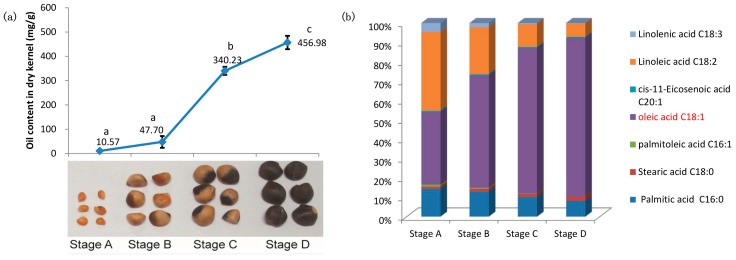
Seed oil contents and compositions. Quantification of total oil contents (**a**) and FA components (**b**) in *C. oleifera* seeds at four development stages. The mean and standard deviation values were calculated based on three independent measurements. Different lowercase letters of the stages indicate a significant difference at the *p* < 0.05 level according to one-way ANOVA. Stages A, B, C and D were 264, 294, 324 and 354 days after fertilization, respectively. mg/g means oil content (mg)/dry kernel (g). % means the FA accounts for the percent of all FAs.

To reveal the accumulation pattern of seed oil, we extracted and measured the total oil contents of *C. oleifera* seeds at four development stages. We showed that the oil content increased slowly in the early period (Stage A–Stage B) and displayed substantially higher content at Stage C and Stage D (Figure 1a). To further reveal the compositional properties of FAs in *C. oleifera* seed, we performed gas chromatography analyses to measure seed oil FA composition in different development stages. We identified seven main kinds of FAs in total, which accounted for over 99.63% of all FAs at every stage (Figure 1b, Table S1). The 7 FAs included 2 saturated FAs (palmitic and stearic acids, 16:0 and 18:0), 3 MUFAs (palmitoleic, oleic and *cis*-11-eicosenoic acid, 16:1, 18:1 and 20:1) and 2 PUFAs (linoleic and linolenic acid, 18:2 and 18:3). In all stages, the unsaturated FAs content, accounting for 84.23–89.27% of all FAs, were much higher than the saturated FAs (Figure 1b, Table S1). For the four seed development stages, in the saturated FA, 16:0 was more abundant; among MUFAs, 18:1 was most abundant, accounting for 95.79–99.23% of all MUFAs (Figure 1b, Table S1); for the PUFAs, 18:2 was much more abundant than 18:3 (Figure 1b, Table S1).

We showed that contents of five FAs including 16:0, 16:1, 18:1, 18:2 and 18:3 revealed significant difference among the four stages (see Table S1). During the seeds’ development, because the TAG was accumulated quickly, almost all FAs increased in absolute content, and 18:1 increased more than all others. With the seeds’ development, the relative contents of 16:0, 16:1, 18:2 and 18:3 dropped, and the 18:1 relative content was increased greatly (Figure 1b). The ratio of 18:1 in all FAs at Stage D was 2.18-times that at Stage A (Figure 1b, Table S1). Overall, the mature seeds had a higher ratio of unsaturated FA content to saturated FA content and a higher ratio of MUFA content to PUFA content compared with the immature seeds (Figure 1b, Table S1).

### 2.2. RNA-Seq, De Novo Transcriptome Assembly and Functional Annotation

To identify genes associated with oil accumulation, we performed transcriptome sequencing of seeds at different development stages (Stage A, Stage B, Stage C and Stage D) from the ‘Changlin No. 4’ clone. The four libraries generated 8,139,855–8,397,376 total reads. After filtering low-quality reads, a total of 30,940,581 clean reads were obtained, having 91.20–97.11% Q ≥ 20 bases and 46.91–49.03% GC content (see Table S2). Then, these reads of each library were assembled into contigs and unigenes using Short Oligonucleotide Analysis Package (SOAP) de novo separately (see Table S2 and Figure S1). Finally, the unigenes of four libraries were assembled into 77,052 All-Unigenes with a mean length of 640 bp (see Table S2 and Figure S1). We performed a similarity search of All-Unigenes to annotate the transcriptome of *C. oleifera* seeds by BLASTx with an *E*-value less than 10^−5^. We found that there were 51,574 unigenes (64.22%) annotated in the NCBI non-redundant protein (Nr) database, 33,503 unigenes (41.72%) annotated in the Swiss-Prot database, 15,442 unigenes (19.23%) annotated in Clusters of Orthologous Groups (COG) database, 10,493 unigenes (13.07%) annotated in Kyoto Encyclopedia of Genes and Genomes (KEGG) and 7261 unigenes (9.04%) annotated in the GO annotation database (Table 1). A total of 51,725 (64.41%) unigenes were annotated in one or more public databases (Table 1 and Table S3).

Among the unigenes annotated in the GO database, three major categories and 54 subcategories were classified (see Table S4). In the biological process category, the unigenes were distributed into 20 subcategories, and the major subcategories were ‘cellular process’ (GO:0009987, 2634 unigenes) and ‘metabolic process’ (GO:0008152, 2693 unigenes). In the cellular component category, the unigenes were distributed into 11 subcategories, and ‘cell’ (GO:0005623, 4593 unigenes), ‘cell part’ (GO:0044464, 4592 unigenes) and ‘organelle’ (GO:0043226, 3448 unigenes) were the dominant subcategories. In the molecular function category, unigenes were distributed into 11 subcategories. ‘Binding’ (GO:0005488, 3247 unigenes) and ‘catalytic’ (GO:0003824, 2856 unigenes) were the largest subcategories (see Table S4).

### 2.3. Identification of DEGs and GO Enrichment Analysis during Seed Development

**Figure 2:**
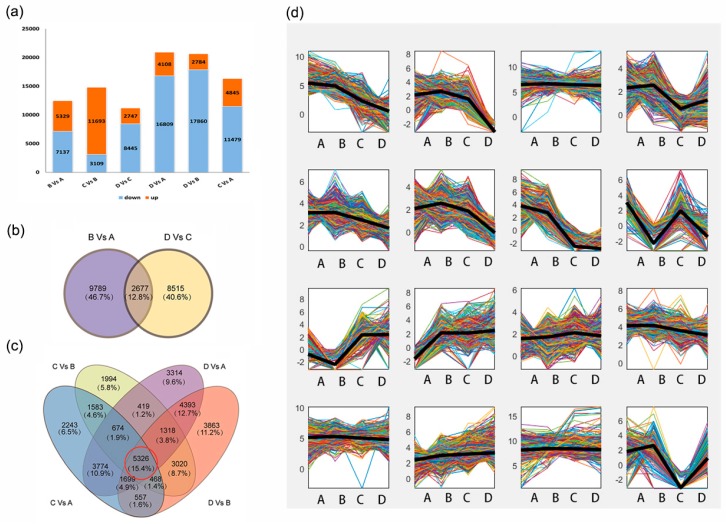
Distribution of DEGs among different development stages in *C. oleifera* seed and clustering of global gene expression. (**a**) The number of up- and down-regulated DEGs between Stages; (**b**) a Venn diagram of DEGs of Stage B-A and Stage C-D; (**c**) a Venn diagram of DEGs of Stage C-A, Stage C-B, Stage D-A and Stage D-B; the red circle indicates the common DEGs; (**d**) clusters of the expressed genes obtained by *K*-means clustering.

To investigate the genes associated with lipid accumulation and FA components, we compared the gene expression levels of the unigene dataset and identified significant DEGs (FDR < 0.05) among different samples. We found there were 12,466 DEGs in the Stage B-A comparison, which included 5329 upregulated genes and 7137 downregulated genes (Figure 2a, Table S5). There were 11,192 DEGs between Stage D-C, including 2747 upregulated genes and 8445 downregulated genes (Figure 2a, Table S5). Stage B-A and Stage D-C had comparable numbers of DEGs, but only a small proportion of DEGs were shared (2677 genes 12.8%, Figure 2b). We showed that Stage C-A and Stage C-B had more common DEGs (Figure 2a,c, Table S5), which resembled the scenario of Stage D-A and Stage D-B (Figure 2c, Table S5). The distribution of DEGs between stages of high and low oil contents revealed that 5326 DEGs (15.4% of total DEGs) were shared in Stage C-A, Stage C-B, Stage D-A and Stage D-B (Figure 2c). These results indicated that there were substantial alterations of gene expression among different development stages in *C. oleifera* seeds. It is possible that the changes of common DEGs among stages were associated with the oil accumulation pattern.

We performed the clustering analysis of the expressed genes with a total reads per kilobases per million reads (RPKM) value over 10 using *K*-means; the results showed that there were 16 expression level changing patterns in all unigenes, and clusters 9 and 10 might be related to TAG synthesis (Figure 2d, Table S6).

**Figure 3:**
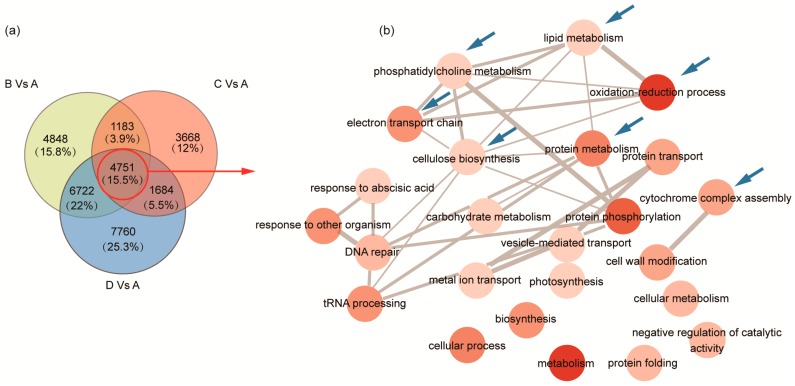
GO enrichment analysis of common DEGs. (**a**) A Venn diagram of DEGs in Stage B-A, Stage C-A and Stage D-A; the red circle indicates the common DEGs in Stage B-A, Stage C-A and Stage D-A; (**b**) common unigenes (4751) were used for GO enrichment analysis and grouped by ReviGO. The darker the red color indicates a smaller FDR-corrected *p*-value. Blue arrows indicate relevant biological process of lipid metabolism and their direct connections.

The oil of *C. oleifera* seed mostly accumulated at the period from Stage B–Stage C (Figure 1a). To evaluate the functional properties of DEGs, We performed the GO enrichment analysis of DEGs during the Stage B-C transition. The results showed that 152 and 308 significantly-enriched GO terms were identified (FDR < 0.001) in down- and up-regulated genes of Stage B-C, respectively (Table S7 and Figure S2). Additionally, there were a few GO terms related to FA synthesis or TAG accumulation overrepresented in the downregulated genes (Figure S2a). In the upregulated genes, many GO terms related to multiple biological process were overrepresented, such as ‘lipid metabolism’, ‘mannose biosynthesis’, ‘terpenoid biosynthesis’, ‘cellular metabolism’, ‘carbohydrate metabolism’, ‘nitrogen compound metabolism’, etc. (Figure S2b). We further performed GO enrichment analysis of the common DEGs of Stage B-A, Stage C-A and Stage D-A (Figure 3a, Table S5), and 138 significantly-enriched GO terms were identified (FDR < 0.001) (see Table S8). In the biological process, multiple GO terms were significantly overrepresented, which included some GO terms relative to FA synthesis, oil accumulation and seed development, such as ‘lipid metabolism’, ‘phosphatidylcholine metabolism’, ‘oxidation-reduction process’, ‘protein metabolism’, ‘cytochrome complex assembly’, ‘electron transport chain’, ‘cellulose biosynthesis’, etc. The GO terms were grouped to reveal the biological processes related to oil accumulation and seed development (Figure 3b). The results indicated that multiple biological pathways, including ‘lipid metabolism’, ‘phosphatidylcholine metabolism’, ‘oxidation-reduction process’, ‘cytochrome complex assembly’ etc., might be involved in FA synthesis and oil accumulation in *C. oleifera*.

### 2.4. Identification and Expression Profiling of Enzymes Involved in Lipid Biosynthesis

**Figure 4:**
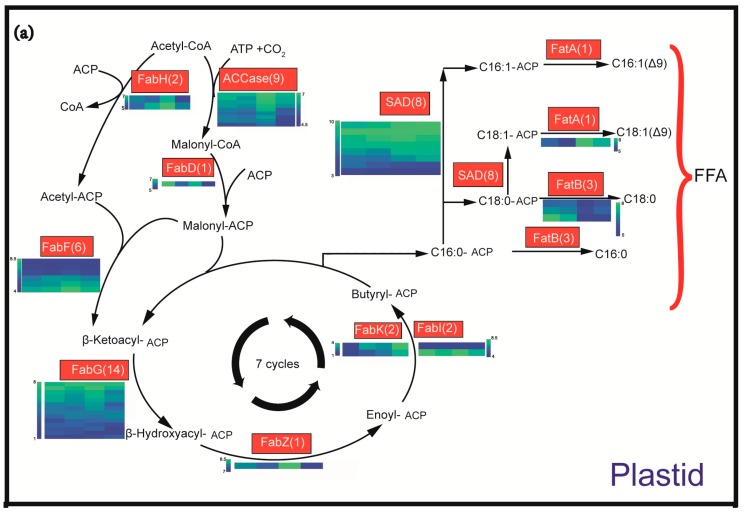

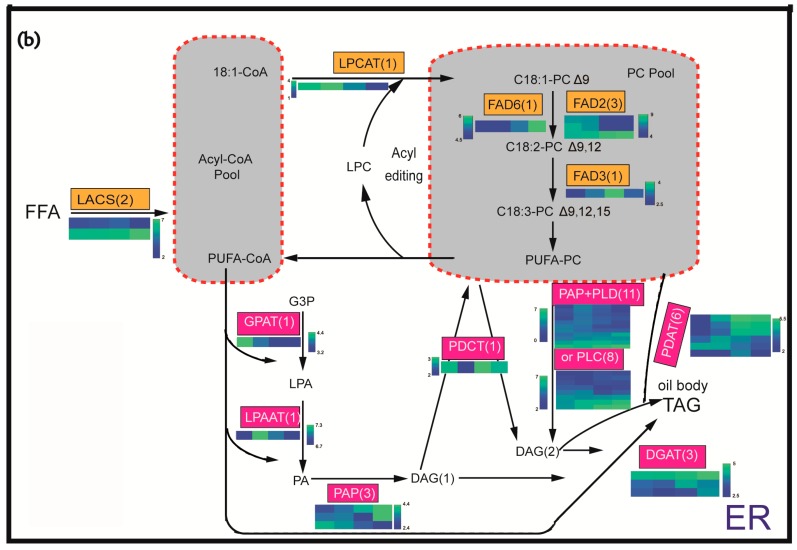
Heatmap plots of genes of fatty acid biosynthesis pathway *C. oleifera*. The number in parentheses means the number of unigenes encoding these enzymes, which had a total RPKM value over 20. Plastid FA synthesis in the upper part (**a**); ER FA modification, acyl editing and TAG assembly in the lower part (**b**). The red boxes are the enzymes related to FA synthesis; the yellow boxes are the enzymes related to FA modification and acyl editing; the purple boxes are the enzymes related to TAG assembly. acyl-CoA: diacylglycerol (DAG) (1) was de novo synthesized DAG, and DAG (2) was PC-derived DAG. Data for the relative expression levels of genes were obtained by using the log2 of RPKM values. Abbreviations: ACCase, acetyl-CoA carboxylase; ACP, acyl carrier protein; DAG, diacylglycerol; DGAT, acyl-CoA:DAG acyltransferase; FabD, malonyl CoA:ACP transacylase; FabF, β-ketoacyl-ACP synthase; FabG, β-ketoacyl-ACP reductase; FabH, acetyl CoA:ACP transacylase; FabI and FabK, enoyl-ACP reductase; FabZ, β-hydroxyacyl ACP dehydrase; FAD2, fatty acid desaturase 2 (Δ-12 desaturase); FAD3, FAD7 and FAD8, Δ-15 desaturase; FAD6, fatty acid desaturase 6 (Δ-12 desaturase); FatA and FatB, acyl-ACP thioesterase; FFA, free fatty acid; G3P, glycerol-3-phosphate; GPAT, acyl-CoA:G3P acyltransferase; LACS, long chain acyl-CoA synthetase; LPA, lyso-phosphatidic acid; LPAAT, acyl-CoA:LPA acyltransferase; LPCAT, acyl-CoA:lysophosphatidylcholine acyltransferase; PA, phosphatidic acid; PAP, PA phosphatase; PC, phosphatidylcholine; PDAT, phospholipid:diacylglycerol acyltransferase; PDCT, PC:DAG cholinephosphotransferase; PLC, phospholipase C; PLD, phospholipase D. PUFA, polyunsaturated fatty acid; SAD, stearoyl-ACP desaturase (Δ-9 desaturase); TAG, triacylglycerol; ER, endoplasmic reticulum.

To investigate the regulatory mechanism of oil accumulation, the plant lipid biosynthesis pathway [6] was evaluated with genes identified in the *C. oleifera* transcriptome. We found 385 unigenes associated with the biosynthesis of oil based on the annotation (see Table S9) and enzymes at each step had one or more corresponding unigenes (Figure 4). We performed the clustering analysis of these genes using Short Time-series Expression Miner (STEM) clustering, and the results showed that the expression of these genes was divided into six changing patterns (Figure S3). Most genes belong to three changing patterns, persistently downregulated, expression peak at Stage B and C, and persistently upregulated during the seed development (Figure S3 and Table S10).

Among 385 unigenes, 66 unigenes (FAs synthase genes) were involved in the FAs biosynthesis pathway in plastid (Figure 4a and Table S11); 48 unigenes were involved in the FA modification and acyl editing pathway (Figure 4b and Table S12); 108 unigenes were associated with the TAG assembly (Figure 4b and Table S13). To understand the potential roles of lipid biosynthesis enzymes, the expression of unigenes with the sum RPKM value over 20 were analyzed further (Figure 4). We showed that some homologous transcripts displayed similar expression patterns, for example the acetyl CoA:ACP transacylase and ACCase transcripts had similar expression profiles to one another during seed development (Figure 4a). On the other hand, some transcripts of several gene families had differential or reversed expression patterns over seed development. For instance, the levels of *β-ketoacyl-ACP reductase*, *SADs*, *FAD2* and *phospholipid:diacylglycerol acyltransferase* (*PDATs*) were peaked at different stages (Figure 4), which suggested the function study of every member in a gene family might be important in lipid biosynthesis.

To investigate the high-level 18:1 accumulation in *C. oleifera* seeds, we focused on several key enzymes related to 18:1 biosynthesis. The SADs catalyze the 18:0-ACP to 18:1-ACP, which had eight homologous transcripts with the sum RPKM over 20 (Figure 4a). Six transcripts of *SADs* had peak expressions at Stage C or D, and two transcripts peaked at Stage A (Figure 4a). FAD2 and fatty acid desaturase 6 desaturate 18:1-phosphatidylcholine (PC) to 18:2-PC, which will reduce the 18:1 contents of TAG (Figure 4b). Notably, we found that two transcripts of *FAD2* had inverted expression patterns with most *SADs*, which were dramatically downregulated at Stage C and Stage D (Figure 4). These expression profiles of enzymes correlated well with the oil content and FA compositions during the seed development, suggesting a synergistic regulatory mechanism involved in fine-tuning the seed oil quality.

TAG assembly is an important step for oil body synthesis and accumulation in seed. We found 33 transcripts including 11 *phospholipase D* (*PLD*), 8 *phospholipase C* (*PLC*), 6 *PDAT*, *3 phosphatidic acid phosphatase* (*PAP*), 3 *DGAT*, 1 *acyl-CoA:glycerol-3-phosphate acyltransferase* (*GPAT*) and 1 *acyl-CoA:lyso-phosphatidic acid acyltransferase* (*LPAAT*) that were expressed at high levels in seeds (Figure 4b). *GPAT* (All-Unigene63667) and *LPAAT* (All-Unigene29697) had the highest expression levels at Stage A and Stage B, respectively. *Acyl-CoA:lysophosphatidylcholine acyltransferase* (*LPCAT*) (All-Unigene17565), the main enzyme of “acyl editing”, had the highest expression levels at Stage B (Figure 4b).

DGAT is identified as a rate-limiting enzyme to catalyze the final step of oil body formation in several oil crops [19,20,21]. We found that the three transcripts corresponding to DGATs consistently peaked at the late stages in *C. oleifera* seeds (Figure 4b). These results indicated that the regulation of *DGAT* expression is an important regulator of TAG assembly and oil accumulation in *C. oleifera*, which is the same as other oil crops.

### 2.5. Validation of RNA-Seq Results by qRT-PCR

**Figure 5:**
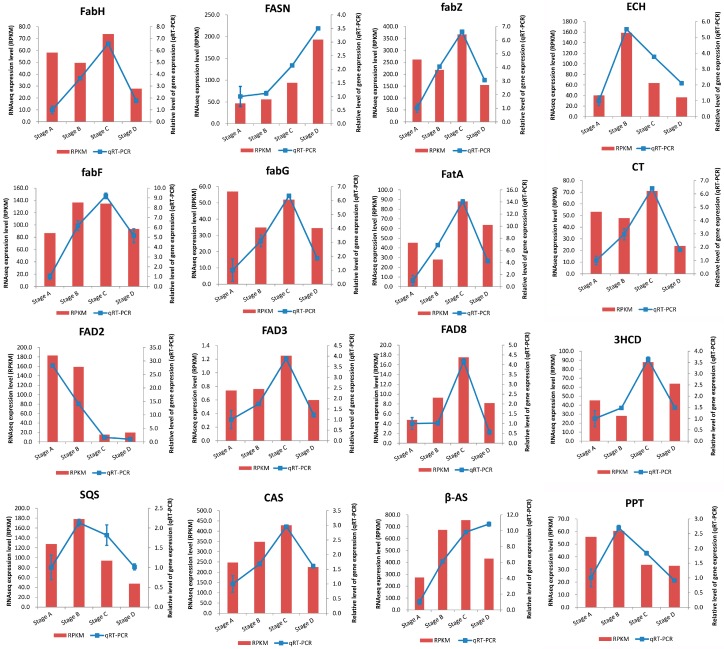
Temporal changes in transcriptional levels for 16 candidate genes in *C. oleifera* seeds. Relative expression levels of qRT-PCR calculated using *CESA* as the reference gene are shown in the right *y*-axis. RNA-Seq expression of the transcript (RPKM) is shown in the left *y*-axis.

To confirm the accuracy and reproducibility of the RNA-Seq results, we selected 16 candidate genes for qRT-PCR analysis (Figure 5). Among 16 candidate genes, 6 candidate genes were involved in the FA synthesis pathway including *acetyl CoA:ACP transacylase* (All-Unigene29235), *FA synthase* (All-Unigene11731), *β-ketoacyl-ACP synthase* (All-Unigene25953), *β-ketoacyl-ACP reductase* (All-Unigene2917), *β-Hydroxyacyl ACP dehydrase* (All-Unigene5873), *acyl-ACP thioesterase A* (*FatA*, All-Unigene26324) and 3 candidate genes involved in the FA desaturation pathway, including *FAD2* (All-Unigene25975), *Δ-15 desaturase 3* (All-Unigene1225) and *Δ-15 desaturase 8* (All-Unigene7225). Three candidate genes of *squalene synthetase* (All-Unigene19917), *squalene cyclase* (All-Unigene20104) and *β-amyrin synthase* (All-Unigene28534) were involved in steroid biosynthesis. The remaining four candidate genes encoding *3-hydroxyacyl-CoA dehydrogenase* (All-Unigene27244), *acetyl-CoA carboxylase carboxyl transferase* α subunit (All-Unigene12649), *enoyl-CoA hydratase* (All-Unigene29899) and *palmitoyl-protein thioesterase* (All-Unigene6679) were involved in FA catabolism. The expression levels of each gene in Stages A, B, C and D were measured through qRT-PCR and compared with the RNA-Seq results (Figure 5). We showed that the expression profiles of the 16 unigenes were mostly consistent between RT-PCR and RNA-Seq experiments (Figure 5), which indicated that the expression profiles of our transcriptomics results were highly confident.

## 3. Discussion

### 3.1. Oil Content and FA Composition Correlated with Expression Profiles of Lipid Biosynthesis Genes during the Seed Development of C. oleifera

*Camellia* oil is called ‘oriental olive oil’ due to the high contents of unsaturated FAs [1,28,34]. We measured the dry kernel oil content and FA composition and found that the oil contents had a significant difference during seed development (Figure 1). The lipid accumulation rate was slower at the early period and quicker at the late stages (Figure 1a), which was consistent with the previous results [31]. The significant change of oil content and FA composition suggested that some key genes had expression level regulation during the seed development of *C. oleifera*. The transcriptomics analysis identified six subsets of up- and down-regulated genes among stages (Figure 2). We showed that there were many more DEGs across the stages with significant changes of oil contents than DEGs between the early (Stage B-A) and late stages (Stage D-C). More importantly, the DEGs between the stages with significant differences of oil contents were shared in a large proportion (Figure 2). These results indicate that the substantial changes of oil contents and FA compositions are regulated by some key pathways, and DEGs discovered in this work will be useful for identifying regulators of lipid biosynthesis.

We evaluated the lipid biosynthesis pathway to investigate gene expression patterns of key enzymes in *C. oleifera* (Figure 4). Previous studies revealed that the levels of saturated FAs and carbon chain length are determined in the plastid FA synthesis pathway [6]. ACCase is thought to be a key enzyme controlling the flux of carbon into FAs [35,36]. In this study, nine *ACCases* were identified, and all of them had a similar expression trend peaked at Stage C (Figure 4a, Table S9), which was consistent with the oil accumulation trend (Figure 1a). It suggested that ACCase and the majority of enzymes related to FA synthesis may be the important enzymes related to oil accumulation in *C. oleifera*. It is demonstrated that the FA carbon chain length and saturation are regulated by the activities of *FatA*, *Acyl-ACP thioesterase B* (*FatB*), *SAD* and *KASII* [37,38]. In this study, we showed that 1 *FatA*, 3 *FatB* and 8 *SADs* were differentially expressed (Figure 4a). During the seed development, *FatA* had the highest expression level at Stage C, which was consistent with the oleic acid content quickly raised at Stage C (Figure 1b, Table S1). The expression level of *FatB* was downregulated with the seed ripening, which was consistent with the downtrend of total saturated FA content (Figure 1b). These results suggest that the expression of FA biosynthesis genes, such as *FatA*, *FatB* and *SADs*, is regulated in a synergistic manner to direct the carbon source for oil production in *C. oleifera* seeds.

### 3.2. A Synergistic Regulation of SADs and FAD2 Contributing to the High 18:1 FA Content

The 18:1 FA is dominant in *Camellia* oil, which resembles the study in hickory [23]. FatA and FatB are the enzymes that hydrolyze the 18:1-ACP and 16:0-ACP to produce free 18:1 and 16:0, respectively; and SADs catalyze 18:0-ACP to produce 18:1-ACP, which is the rate-limiting step in the formation of unsaturated FAs [6]. To achieve the high level of 18:1, we showed that the upregulation of *SADs* coupled with the downregulation of *FAD2* might be critical in *Camellia* oil biosynthesis (Figure 4). The transcriptomic and lipid profiling in hickory seeds (*Carya cathayensis*) proposed that synergistic regulation of a high level of *SAD* with a low level of *FAD2* facilitated the 18:1 accumulation [23]. Our results are in good agreement with the discovery in hickory, which suggests that perhaps a similar regulatory mechanism is involved. In the model plant *Arabidopsis*, the transcription factors, such as *WRINKLED1*, *LEAFY COTYLEDON2* and *FUSCA3*, are revealed as master regulators to control a subset of enzyme genes to modify lipid biosynthesis [39]. We identified the *WRINKLED1* and *FUSCA3* homologs from *C. oleifera* transcriptome with an *E*-value less than 10^−5^ and found that the expression level of the *WRINKLED1* homolog (All-Unigene10602) had no significant difference between the four stages. Multiple differentially-expressed *FUSCA3* unigenes were detected, including All-Unigene27710, All-Unigene1623, All-Unigene38005, All-Unigene3928, All-Unigene19081, All-Unigene14850 and All-Unigene1408, with a consistent downregulation trend during the seed development. It was shown that the *FUSCA3* might be an important regulator to modify lipid biosynthesis in *C. oleifera*. We could not found any homolog transcript of *LEAFY COTYLEDON2*. A recent study in olive tree has demonstrated that the evolution of a siRNA from a transposable element negatively regulated the expression of *FAD2* during seed oil biosynthesis, which was correlated with the high content of 18:1 FAs [26]. These results suggested a potential convergent evolution process regarding the accumulation of 18:1 FA in different oil crops. However, future studies are needed to understand how *SADs* and *FAD2* expression levels are regulated in *C. oleifera*.

### 3.3. Utilization of DAG Production by PLD or PLC Plus PAP as the Substrate for TAG Synthesis May Be the Important Pathway of the Incorporation of FA PC-Derived in TAG in C. oleifera

The incorporation of FA PC-derived in TAG may involve three mechanisms: (1) “acyl editing” [6,40]; (2) transfer of an FA from PC to DAG, producing TAG by PDAT [6,41]; (3) TAG synthesis using PC-derived DAG as the substrate [6,41]. LPCAT isoforms can play a role in the exchange of FA between the pool of PC and that of acyl-CoA through the “acyl editing” mechanism [40]. This process constitutes the first potential mechanisms for FA PC-derived incorporation into TAG. Because *LPCAT* (All-Unigene17565) was downregulated during the seed development (Figure 4b), “acyl editing” was not likely to play an important role during *Camellia* oil accumulation. A second possible mechanism consists of the transfer of FA from PC to DAG by the PDAT to form lyso-PC and TAG. LPCAT is then necessary to regenerate PC from lyso-PC produced by PDAT. Therefore, even though the three main *PDAT* unigenes (All-Unigene6121, All-Unigene63539, All-Unigene73309) were upregulated at Stage C (Table S13), downregulation of *LPCAT* might be sufficient to lower FA incorporation in TAG by this mechanism during *Camellia* oil biosynthesis. The third mechanism has two alternative enzymatic routes to generate PC-derived DAG. The first possible route involves DAG-PC inter-conversion. DAG species formed through the Kennedy pathway are converted to PC through CPT or PDCT activity [6,42]. Oleate esterified to the resulting PC at the sn-2 position can then be desaturated to 18:2 by FAD2, and the resulting 18:2 can be further desaturated to 18:3 by FAD3. The reversibility of the reactions catalyzed by CPT and PDCT enables enrichment of DAG in PUFA. Another possible route is a lipase-based mechanism utilizing PLC or PLD plus PAP. Different plants have different combinations of PC-derived DGA generation routes [43,44]. In this study, we found one transcript of PDCT with a low expression level and could not find any transcript of CPT, which was likely to contribute to the very high *Camellia* oil content in MUFA. We obtained 11 *PLD* and eight *PLC* homologous transcripts with the sum RPKM over 20, respectively. Additionally, the main *PLD* and *PLC* unigenes were upregulated at the late stage (Table S13). It was possible that the PLC or PLD plus PAP enzymatic route was the more important PC-derived DAG generation pathway in *C. oleifera*, which resembled the study in soybean [44].

### 3.4. DGATs May Be Rate-Limiting Genes in the Control of the TAG Flux and Oil Production

In TAG synthesis, DAG is synthesized by two main pathways in plants: (1) de novo DAG synthesis (Kennedy pathway) and (2) conversion of the membrane lipid PC to DAG [41]. The choice of de novo synthesis and PC-derived DAG appears to be diverse and influenced by species, tissues, developmental stages or environmental conditions [40,41,45]. It is shown that most 18:1 entered PC-derived DAG synthesis, but not de novo DAG synthesis in many plants [37].

In this study, transcripts involved in both de novo and PC-derived DAG synthesis were found (Figure 4b, Table S9). We examined the expression profiles of these transcripts and found that the *GPAT* and *LPAAT* involved in the Kennedy pathway showed downregulation (Figure 4b). Most of the *phospholipase C* and *PDAT* in PC-derived DAG synthesis were upregulated during seed ripening (Figure 4b). Although the 18:1 (which was not modified upon PC) content in the accumulated TAG raised from 37.43%–81.53%, and 18:2 (modified upon PC) content decreased from 40.62%–6.80% (Figure 1b, Table S1) during the seed development; the relative flux of TAG synthesis from PC-derived DAG may be more important than that from de novo DAG in *C. oleifera* seed. These results were consistent with the results of GO enrichment analysis in which PC metabolism was significantly enriched when using the common DEGs from Stage B-A, Stage C-A and Stage D-A (Figure 3). DGAT is the last enzyme participating in TAG synthesis pathway and has been shown to play a key role in catalyzing TAG production in many oil crops [20,46,47,48]. We found that three *DGAT* unigenes showed consistent expression profiles peaked at Stage C or Stage D in this study, which were consistent with the oil accumulation trend (Figure 1a). It suggests that *DGAT* may be also playing important roles in the oil accumulation of *C. oleifera* seeds.

## 4. Materials and Methods

### 4.1. Plant Materials

*C. oleifera* var. ‘Changlin No. 4’ is an elite cultivar of oil production in China. In this study, *C. oleifera* ‘Changlin No. 4’ individuals were obtained from a 4-year-old clonal plantation located in Jinhua city, Zhejiang province, China. *C. oleifera* flowers in late October, and the fruits take 12 months to mature. According to the multiyear data of phenotype, *C. oleifera* flowers and fertilizes in late October, and the fruit development is in dormancy until the next May. The fruit begins to expand in May; the seeds expand and the kernels can be isolated until late June. Therefore, all sampling was done in the last third period of seed development in this study. We marked 100 flowers opening on the same day on one tree, and the seeds were harvested at four development stages from the marked fruits, 264 days after fertilization, which was the kernel expanding early period (Stage A), 294 days after fertilization the kernel quickly expanding period (Stage B), 324 days after fertilization the kernel expanding late period (Stage C) and 354 days after fertilization the kernel maturation period (Stage D). One part of the kernels was immediately immersed in liquid nitrogen for about 5 min and then stored at −80 °C in freezers for RNA extraction. The other part of kernels was dried at 105 °C for chemical analysis of oil content and FAs’ compositions.

### 4.2. Oil Content and FA Component Analysis

Total lipid was extracted from kernel by petroleum ether. Kernel oil content was measured by the Soxtec extraction method according to the previous study [49]. FA components were quantified using gas chromatography as described [50]. Data Processing System (DPS 14.50) software [51] was used to perform the statistical analysis. The significant difference among different stages was obtained by one-way ANOVA.

### 4.3. Total RNA Extraction

The total RNAs were extracted from 4 stages of kernels, respectively, using the TRIzol RNA Extraction Kit (Invitrogen, Carlsbad, CA, USA) according to the user manual. RNA quality and quantity were evaluated by 1% agarose gel electrophoresis and an Agilent Bioanalyzer 2100 (Agilent Technologies, Santa Clara, CA, USA) device. The RNAs with a concentration no less than 400 ng/μL, 28S rRNA/18S rRNA over 1.8 and RNA Integrity Number (RIN) over 8.0, were defined as qualified RNA samples for further experiments.

### 4.4. Transcriptome Sequencing, Assembly and Functional Annotation

The 4 μg total RNA from each stage were used for library preparation. mRNA was purified from total RNA using oligo(dT)-attached magnetic beads. The library construction and sequencing was performed by Beijing Genomics Institute (BGI, Shenzhen, China) on an Illumina GAIIx platform according to Trick et al., method [52]. The Illumina GAIIx platform generated short sequencing reads with 75 bp in length by paired-end sequencing. All un-filtered raw data have been submitted to the NCBI BioProject, and the accession number is SRP111395.

Prior to assembly, We implemented a quality filter to remove the adaptor sequences, empty reads and low-quality sequences by controlling the percentage of the nucleotides that had Phred quality scores less than 20 [53]. The reads of the 4 stages were separately assembled into contigs, then unigenes using SOAP de novo software [54]. The unigenes of the 4 stages were further assembled using sequence clustering software TGICL [55] and the redundant sequences removed. The longest and non-redundant unigenes were defined as All-Unigenes in this study.

Functional annotation of All-Unigenes was carried out by BLASTx search with several public databases: the NCBI Nr database, the Swiss-Prot database, the Clusters of Orthologous Groups (COG) database, the Kyoto Encyclopedia of Genes and Genomes (KEGG) and the Gene Ontology (GO) annotation database. The threshold of the *E*-value was less than 10^−5^. After functional annotation, the unigenes with the GO annotation were GO classified using WEGO software [56].

### 4.5. Clustering, Identification of DEGs and GO Enrichment

We performed gene expression clustering by *K*-means and Short Time-series Expression Miner (STEM) methods. The read counts were normalized to the RPKM value to represent the gene expression level. The unigenes with a total RPKM value less than 10 were filtered, and the remaining expression levels were normalized by the log2 method. The MATLAB Bioinformatics toolbox was used to perform *K*-means clustering with Euclidean distance, and STEM clustering was performed as described [57]. DEGs were analyzed using Audic and Claverie’s method [58], and *p*-value correction was performed using Benjamini and Yekutieli’s approach to control FDR. Genes with FDR ≤ 0.001 and RPKM value differences over two-times were regarded as DEG. The DEG distribution among different stages was performed using Venny [59]. GO term enrichment analysis was carried out by the hypergeometric test and visualized using ReviGO [60]. GO terms with an adjusted FDR < 0.0001 by Benjamini and Hochberg’s approach were selected for visualization.

### 4.6. Quantitative RT-PCR Analysis

The RNA samples used for qRT-PCR analysis were the same as those for the next-generation sequencing experiments. Single-strand cDNA for each sample was synthesized using the First-Strand Synthesis System (Invitrogen, Carlsbad, CA, USA). The primer pairs (see Table S14) were designed according to the selected unigene sequences using Primer 5.0 software. The primers were 19–21 bp in length, and had amplicon lengths of 200–260 bp. *Cellulose synthase A* (*CESA*) was used as the reference gene, and the *M*-value was calculated according Zhou et al., method [61] was 0.6265 in this study. The amplification procedures were performed as described [61]. The qRT-PCR was carried out in three replications for each sample, and the relative expression level of genes was calculated by the 2^−ΔΔ*C*t^ method [62].

## 5. Conclusions

In this study, the oil contents and FA compositions of *C. oleifera* seed at different development stages were measured. We showed that total oil production of kernel increased dramatically at the late stages, and 18:1 was predominantly accumulated during seed maturation. Through transcriptomics profiling, we obtained several sets of DEGs between the different seed development stages. We showed that the number of DEGs correlated with the oil accumulation, and more common DEGs were identified across early and late stages of seed development. Further functional enrichment of subsets of DEGs revealed that the lipid biosynthesis pathway and related biological processes were related to seed oil accumulation. Moreover, gene expression analysis of key enzymes suggested that the coordination of high *SAD*s and low *FAD*2 activities was required for 18:1 domination. Overall, our study provides some first hypotheses about and initial clues into the gene expression patterns for oil accumulation and FA composition in *C. oleifera* seed and may pave the way to expedite the breeding of *C. oleifera* with higher oil content and better oil quality.

## Figures and Tables

**Table 1:** Functional annotation of the *C. oleifera* transcriptome.

Annotated Databases	Number of Unigenes	Percentage (%)	≥300 bp	≥1000 bp
Nr Annotation	51,574	64.22	40,908	13,684
Swiss-Prot Annotation	33,503	41.72	28,215	11,410
COG Annotation	15,442	19.23	13,883	6721
KEGG Annotation	10,493	13.07	8551	3564
GO Annotation	7261	9.04	5545	2393
Total	51,725	64.41	40,982	13,689

## Supplementary Materials

Supplementary materials can be found at www.mdpi.com/1422-0067/19/1/108/s1.

## Abbreviations

COG	Clusters of Orthologous Groups
KEGG	Kyoto Encyclopedia of Genes and Genomes
GO	Gene Ontology
PDAT	Phospholipid:diacylglycerol acyltransferase
FatA and FatB	Acyl-ACP thioesterase
PC	Phosphatidylcholine
GPAT	Acyl-CoA:glycerol-3-phosphate acyltransferase
LPAAT	Acyl-CoA:lyso-phosphatidic acid acyltransferase
PUFA	Polyunsaturated fatty acid
ER	Endoplasmic reticulum
FabH	Acetyl CoA:ACP transacylase
FabD	Malonyl CoA:ACP transacylase
FabF	β-Ketoacyl-ACP synthase
FabG	β-Ketoacyl-ACP reductase
FabZ	β-Hydroxyacyl ACP dehydrase
FabI and FabK	Enoyl-ACP reductase
FFA	Free fatty acid
LACS	Long chain acyl-CoA synthetase
LPCAT	Acyl-CoA:LPC acyltransferase
LPC	Lysophosphatidylcholine
FAD	Fatty acid desaturase
G3P	Glycerol-3-phosphate
GPAT	Acyl-CoA:G3P acyltransferase
LPA	Lyso-phosphatidic acid
PA	Phosphatidic acid
PAP	PA phosphatase
PLC	Phospholipase C
PLD	Phospholipase D
