# Supplementary material for: Seed Transcriptomics Analysis in Camellia oleifera Uncovers Genes Associated with Oil Content and Fatty Acid Composition
Source: Int J Mol Sci. 2018 Jan 2;19(1):118. doi: 10.3390/ijms19010118 (PMC5796067; doi:10.3390/ijms19010118)
Supplement: Supplementary file 1 [file ijms-19-00118-s001.zip › Supplementary_v2/Additional File17_Table S14 The list of primers for qRT- PCR.docx]

**Table S10** Primers for qRT- PCR.

| Unigene ID | Gene symbl | Primer sequence(5’→3’) (forword/reverse) |
| --- | --- | --- |
| Unigene19917 | SQS | F: TTTCGCCCTCGTAATTCAAC R: CATGAAAAATGCCAGTCACG |
| Unigene28534 | β-AS | F: GTTGCACCCTGACCAATCGAA R: TCCACATAGAAGGGCACAGCA |
| Unigene20104 | CAS | F: TGGCACATGGTTCGGGGTAAA R: AATGAGAGCCAGCATAGCCCA |
| Unigene11731 | FasN | F: GCTGGAGCTGTCCTCTTGAT R: GCAAATGCCTCATTTATCTCG |
| Unigene29235 | FabH | F: TGCCTGATGGAGGAGTAACC R: GCACAGTGATGGTGATGGTC |
| Unigene25953 | FabF | F: GGGAGAAGGAGCTGGAGTTT R: CTGGTGTGGATGTAGCATGG |
| Unigene2917 | FabG | F: TGCAAAAGCAGGAGTGATTG R: GGTGAAAACCTGTCCGGTAA |
| Unigene5873 | FabZ | F: CAGATTCCGGAAGCCAGTTA R: GGTTGCCTCAGGTTTTGCTA |
| Unigene26324 | FatA | F: CACCGCTTTCCCTTTCTTCT R: GCTTCCGGAATCTGACTTTG |
| Unigene27244 | 3HCD | F: CCTGTTGTGGTGGGAAACTG R: GGAGAACGGAATGTGCGATC |
| Unigene29899 | ECH | F: AAATTCGGAGGGTTCCACTT R: GAAAGTGAGGCAGCGAAAAC |
| Unigene6679 | PPT | F: GGGTGCTCCATCACAAAACT R: ATCGGGGAGTTAAACGGTTC |
| Unigene25975 | FAD2 | F: CCCATTGTTTTCGTCGATCT R: TGTGTCATCAAGCCATTGGT |
| Unigene7225 | FAD8 | F: TTCGTGCTGCAATTCCCAAA R: GCTTCCATGGCCACAATCAT |
| Unigene1225 | FAD3 | F: ATGACAAAGGACAGGCCAAC R: TCATGGCACCCATTATCTGA |
| Unigene12649 | CT | F: CCATCCCTACTGCAACCTGT R: TAGTCGCGATTGCAGTGTTC |

Primers were designed based on the sequences of *C.oleifera* transcriptome library by using Primer 5.0.
