# Supplementary material for: Seed Transcriptomics Analysis in Camellia oleifera Uncovers Genes Associated with Oil Content and Fatty Acid Composition
Source: Int J Mol Sci. 2018 Jan 2;19(1):118. doi: 10.3390/ijms19010118 (PMC5796067; doi:10.3390/ijms19010118)
Supplement: Supplementary file 1 [file ijms-19-00118-s001.zip › Supplementary_v2/Additional File2_Table S2 Details of RNA-Seq data and Summary of transcriptome assembly for C. oleifera..docx]

| **Table S2-1** Details of RNA-seq data for *C. oleifera*   \| Samples \| Total Reads \| Total Nucleotides (nt) \| Clean Reads \| Q20 percentage \| N percentage \| GC percentage \| reads mapped to All-Unigenes \| \| --- \| --- \| --- \| --- \| --- \| --- \| --- \| --- \| \| Stage A \| 8,320,068 \| 1,248,010,200 \| 7,568,240 \| 97.02% \| 0.03% \| 47.55% \| 6,927,210 (91.53%) \| \| Stage B \| 8,397,376 \| 1,259,606,400 \| 7,717,002 \| 97.11% \| 0.02% \| 46.91% \| 7,096,555 (91.96%) \| \| Stage C \| 8,385,809 \| 1,257,871,350 \| 7,990,760 \| 94.52% \| 0.06% \| 49.03% \| 7,336,327 (91.81%) \| \| Stage D \| 8,139,855 \| 1,220,978,250 \| 7,664,578 \| 91.20% \| 0.04% \| 48.76% \| 7,009,257 (91.45%) \| |
| --- | --- | --- | --- | --- | --- | --- | --- | --- | --- | --- | --- | --- | --- | --- | --- | --- | --- | --- | --- | --- | --- | --- | --- | --- | --- | --- | --- | --- | --- | --- | --- | --- | --- | --- | --- | --- | --- | --- | --- | --- |

| **Table S2-2** Summary of transcriptome assembly for *C. oleifera*. | | | | | | | | | | |
| --- | --- | --- | --- | --- | --- | --- | --- | --- | --- | --- |
|  | Contigs | | | |  | Unigenes | | | | All-Unigenes |
|  | Stage A | Stage B | Stage C | Stage D |  | Stage A | Stage B | Stage C | Stage D |  |
| Total number | 111,139 | 119,140 | 89,077 | 74,233 |  | 43,461 | 47,932 | 35,589 | 30,022 | 77,052 |
| Total length | 26,473,043 | 28,743,101 | 20,742,724 | 17,711,685 |  | 24,697,318 | 25,813,424 | 19,500,650 | 16,537,701 | 49,320,033 |
| N50 length | 284 | 284 | 274 | 284 |  | 737 | 680 | 709 | 696 | 927 |
| Mean length | 238 | 241 | 233 | 239 |  | 568 | 539 | 548 | 551 | 640 |
|  |  |  |  |  |  |  |  |  |  |  |

The unigenes of Stage A, B, C, D were further assembled and the longest and non-rebundant unigenes were defined as All-Unigenes.
