# Supplementary figures and images for: Seed Transcriptomics Analysis in Camellia oleifera Uncovers Genes Associated with Oil Content and Fatty Acid Composition
Source: Int J Mol Sci. 2018 Jan 2;19(1):118. doi: 10.3390/ijms19010118 (PMC5796067; doi:10.3390/ijms19010118)

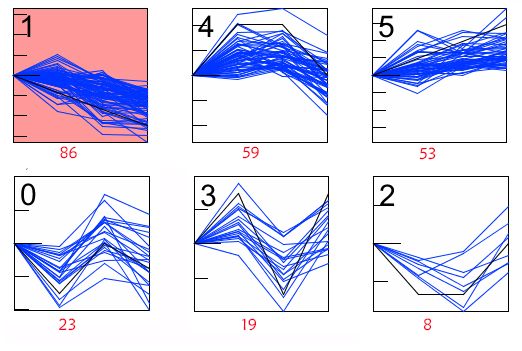

Supplement: Supplementary file 1 [file ijms-19-00118-s001.zip › Supplementary_v2/Additional File12_Fig S3 Clusters of genes related to oil biosynthesis obtained by STEM clustering.jpg]

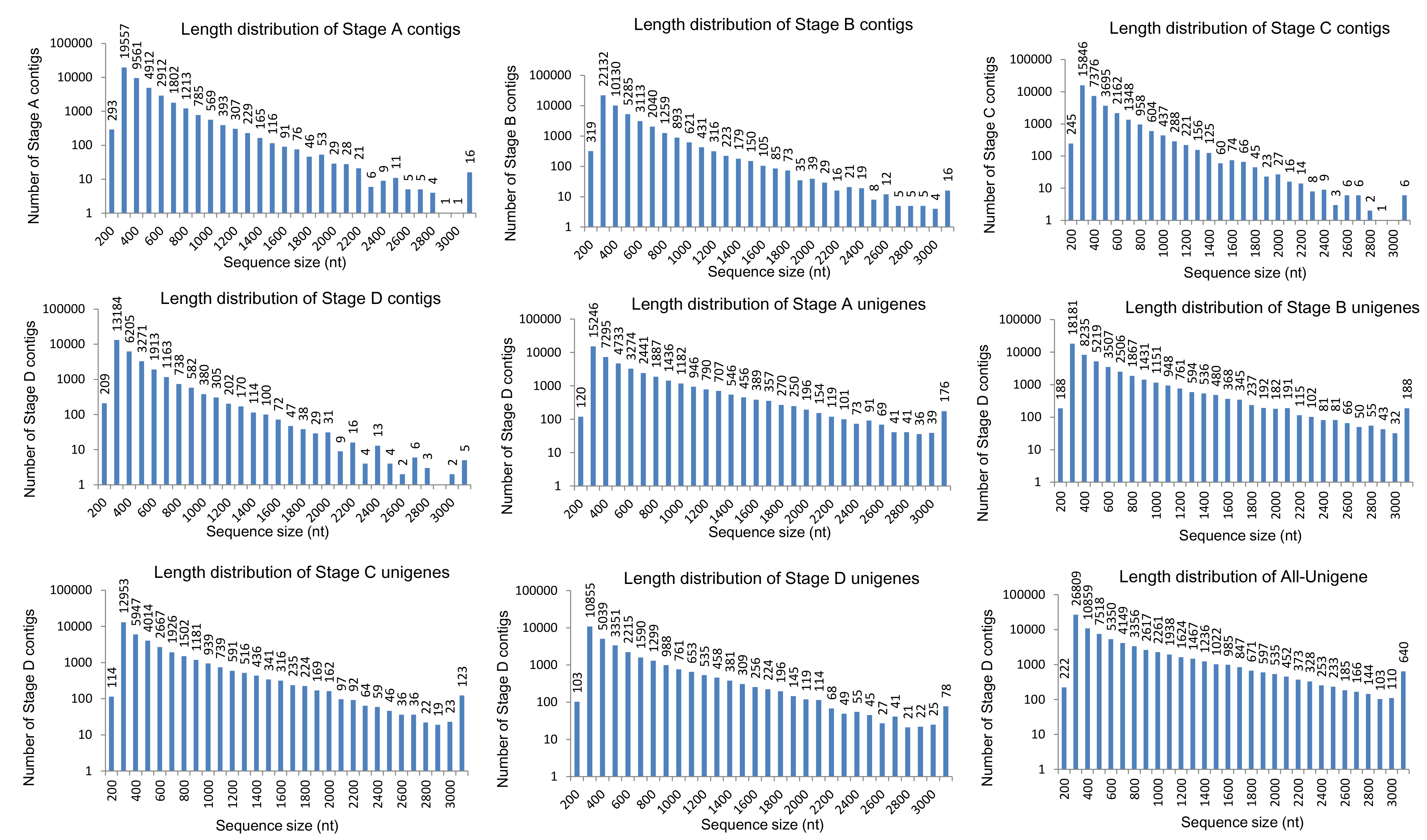

Supplement: Supplementary file 1 [file ijms-19-00118-s001.zip › Supplementary_v2/Additional File3_Fig S1 length distribute of contigs and unigenes.jpg]

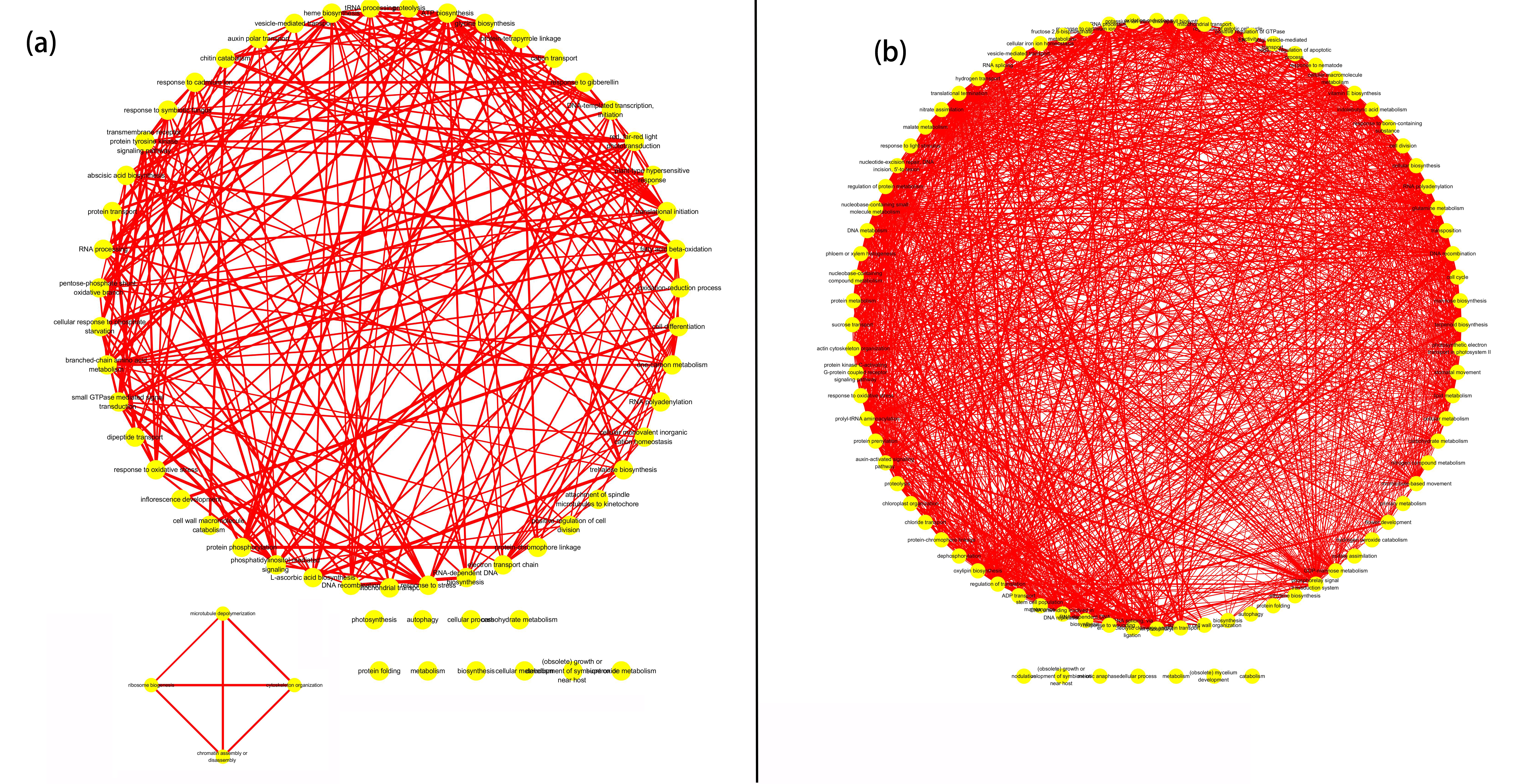

Supplement: Supplementary file 1 [file ijms-19-00118-s001.zip › Supplementary_v2/Additional File9_Fig S2 GO enrichment analysis of DEGs between Stage B and Stage C. .jpg]
